# Tris(2-methoxy­phen­yl)phosphine

**DOI:** 10.1107/S1600536809020595

**Published:** 2009-06-06

**Authors:** Omar bin Shawkataly, Mohd. Aslam A. Pankhi, Imthyaz Ahmed Khan, Chin Sing Yeap, Hoong-Kun Fun

**Affiliations:** a Chemical Sciences Programme, Centre for Distance Education, Universiti Sains Malaysia, 11800 USM, Penang, Malaysia; b X-ray Crystallography Unit, School of Physics, Universiti Sains Malaysia, 11800 USM, Penang, Malaysia

## Abstract

In the title compound, C_21_H_21_O_3_P, the whole mol­ecule is disordered over two sets of positions with refined occupancies of 0.503 (1) and 0.497 (1). The dihedral angles between the three benzene rings are 72.9 (2)°, 82.9 (3)° and 70.0 (2)° in the major disorder component and the corresponding angles in the minor disorder component are 85.0 (2)°, 79.2 (2)° and 72.3 (2)°. The crystal structure is stabilized by C—H⋯π inter­actions.

## Related literature

For P–C bond lengths and C–P–C angles, see: Uttecht *et al.* (2005 ▶). For the stereochemistry of tris­(2-methoxy­phen­yl) phosphine complexes and for P–C bond distances, see: Abbassioun *et al.* (1990 ▶); Shawkataly *et al.* (1996 ▶); Hirsivaara *et al.* (2000 ▶); Barnes *et al.* (2006 ▶); Bott *et al.* (2007 ▶); Romeo *et al.* (2006 ▶). For bond-length data, see: Allen *et al.* (1987 ▶). For the stability of the temperature controller used for the data collection, see: Cosier & Glazer (1986 ▶).
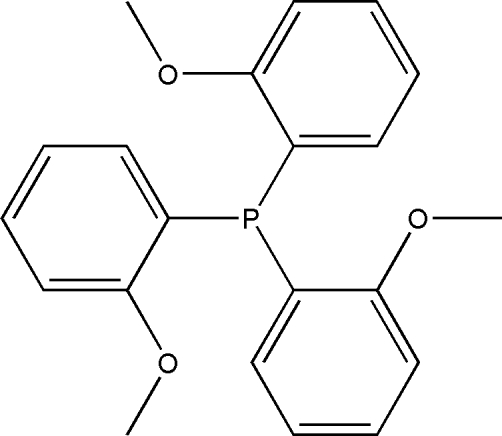


## Experimental

### 

#### Crystal data

C_21_H_21_O_3_P
*M* *_r_* = 352.35
Monoclinic, 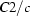

*a* = 29.5721 (4) Å
*b* = 8.2201 (1) Å
*c* = 14.9409 (2) Å
β = 96.381 (1)°
*V* = 3609.42 (8) Å^3^
*Z* = 8
Mo *K*α radiation
μ = 0.17 mm^−1^
*T* = 120 K
0.47 × 0.37 × 0.11 mm

#### Data collection

Bruker SMART APEXII CCD area-detector diffractometer
Absorption correction: multi-scan (**SADABS**; Bruker, 2005 ▶) *T*_min_ = 0.862, *T*_max_ = 0.983
40913 measured reflections
5318 independent reflections
4128 reflections with *I* > 2σ(*I*)
*R*_int_ = 0.032

#### Refinement

*R*[*F* ^2^ > 2σ(*F* ^2^)] = 0.064
*wR*(*F* ^2^) = 0.163
*S* = 1.05
5318 reflections
413 parameters
189 restraints
H-atom parameters constrained
Δρ_max_ = 0.44 e Å^−3^
Δρ_min_ = −0.50 e Å^−3^

### 

Data collection: *APEX2* (Bruker, 2005 ▶); cell refinement: *SAINT* (Bruker, 2005 ▶); data reduction: *SAINT*; program(s) used to solve structure: *SHELXTL* (Sheldrick, 2008 ▶); program(s) used to refine structure: *SHELXTL*; molecular graphics: *SHELXTL*; software used to prepare material for publication: *SHELXTL* and *PLATON* (Spek, 2009 ▶).

## Supplementary Material

Crystal structure: contains datablocks global, I. DOI: 10.1107/S1600536809020595/ci2802sup1.cif

Structure factors: contains datablocks I. DOI: 10.1107/S1600536809020595/ci2802Isup2.hkl

Additional supplementary materials:  crystallographic information; 3D view; checkCIF report

## Figures and Tables

**Table 1:** Hydrogen-bond geometry (Å, °)

*D*—H⋯*A*	*D*—H	H⋯*A*	*D*⋯*A*	*D*—H⋯*A*
C21*A*—H21*C*⋯*Cg*1^i^	0.96	2.83	3.662 (3)	145

Symmetry code: (i) 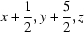. *Cg*1 is the centroid of the C13*A*–C18*A* ring.

**Fig. 1.**
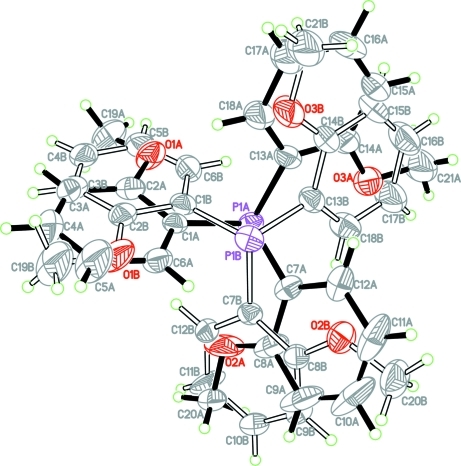
The molecular structure of the title compound with atom labels and 50% probability displacement ellipsoids for non-H atoms. Both disorder components are shown.

**Fig. 2.**
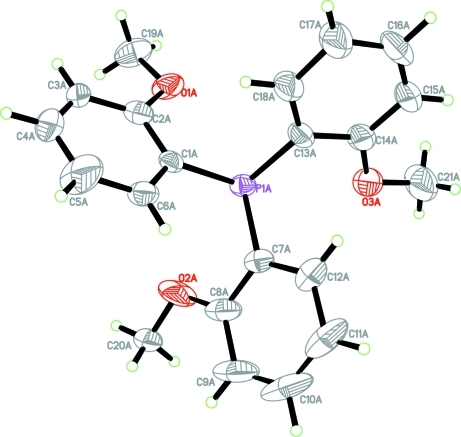
The major disorder component of the title compound with atom labels and 50% probability displacement ellipsoids for non-H atoms.

**Fig. 3.**
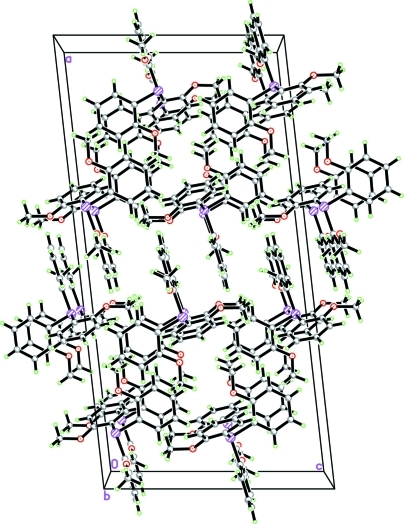
The crystal packing of the title compound, showing molecular stacking down the b axis. Only the major disorder component is shown.

## supplementary crystallographic information

### Comment

The structure determination of the title compound was undertaken as part of a project to study the stereochemistry of substituted triphenylphosphine ligands (Abbassioun *et al.*, 1990; Shawkataly *et al.*, 1996; Hirsivaara *et al.*, 2000; Barnes *et al.*, 2006; Bott *et al.*, 2007). Some of these interesting complexes have been synthesized using this tripodal ligand. Specially, its complex with platinum exhibits fluxionality and has been shown to behave as molecular gears (Romeo *et al.*, 2006). The X-ray crystal structure of its thio analogue namely, tris[2-(methylsulfanyl)phenyl]phosphine, shows two independent molecules (Uttecht *et al.*, 2005).

The whole molecule of title compound is disordered over two positions (Fig. 1 and 2) with refined occupancies of 0.503 (1) and 0.497 (1). The P—C bond lengths and C—P—C angles are comparable to a related structure (Uttecht *et al.*, 2005). Bond lengths (Allen *et al.*, 1987) and angles are within normal ranges. The dihedral angles between the benzene rings C1-C6 (A), C7-C12 (B) and C13-C18 (C) are: A/B 72.9 (2)°, A/C 82.9 (3)° and B/C 70.0 (2)° for the major disorder component, and A/B 85.0 (2)°, A/C 79.2 (2)° and B/C 72.3 (2)° for the minor disorder component.

In the crystal structure, a C18B···C19B(x,1+y,z) contact [3.112 (6) Å], shorter than the sum of the van der Waals radii is observed. The crystal structure (Fig. 3) is stabilized by C—H···π interactions (Table 1).

### Experimental

The title compound was supplied by Strem Chemicals. Single crystals of were obtained by slow evaporation of an ethanol solution.

### Refinement

The whole molecule is disordered over positions, with occupancies of 0.503 (1) and 0.497 (1). The same U^ij^ parameters were used for atom pairs C17A/C17B, C5A/C19B, C2A/C20A and C21B/C21A, and all disordered atoms were subjected to a rigid bond restraint. All H atoms were positioned geometrically and refined using a riding model with C-H = 0.93–0.96 Å and U_iso_(H) = 1.2 and 1.5 U_eq_(C). A rotating-group model was applied for the methyl groups.

### Crystal data

**Table:**

C_21_H_21_O_3_P	*F*(000) = 1488
*M**_r_* = 352.35	*D*_x_ = 1.297 Mg m^−^^3^
Monoclinic, *C*2/*c*	Mo *K*α radiation, λ = 0.71073 Å
Hall symbol: -C 2yc	Cell parameters from 9954 reflections
*a* = 29.5721 (4) Å	θ = 1.4–30.1°
*b* = 8.2201 (1) Å	µ = 0.17 mm^−^^1^
*c* = 14.9409 (2) Å	*T* = 120 K
β = 96.381 (1)°	Block, colourless
*V* = 3609.42 (8) Å^3^	0.47 × 0.37 × 0.11 mm
*Z* = 8	

### Data collection

**Table:**

Bruker SMART APEXII CCD area-detector diffractometer	5318 independent reflections
Radiation source: fine-focus sealed tube	4128 reflections with *I* > 2σ(*I*)
graphite	*R*_int_ = 0.032
φ and ω scans	θ_max_ = 30.1°, θ_min_ = 1.4°
Absorption correction: multi-scan (*SADABS*; Bruker, 2005)	*h* = −41→41
*T*_min_ = 0.862, *T*_max_ = 0.983	*k* = −11→11
40913 measured reflections	*l* = −21→18

### Refinement

**Table:**

Refinement on *F*^2^	Primary atom site location: structure-invariant direct methods
Least-squares matrix: full	Secondary atom site location: difference Fourier map
*R*[*F*^2^ > 2σ(*F*^2^)] = 0.064	Hydrogen site location: inferred from neighbouring sites
*wR*(*F*^2^) = 0.163	H-atom parameters constrained
*S* = 1.05	*w* = 1/[σ^2^(*F*_o_^2^) + (0.0605*P*)^2^ + 3.6006*P*] where *P* = (*F*_o_^2^ + 2*F*_c_^2^)/3
5318 reflections	(Δ/σ)_max_ = 0.001
413 parameters	Δρ_max_ = 0.44 e Å^−^^3^
189 restraints	Δρ_min_ = −0.50 e Å^−^^3^

### Special details

**Table:**

Experimental. The crystal was placed in the cold stream of an Oxford Cyrosystems Cobra open-flow nitrogen cryostat (Cosier & Glazer, 1986) operating at 120.0 (1)K.
Geometry. All esds (except the esd in the dihedral angle between two l.s. planes) are estimated using the full covariance matrix. The cell esds are taken into account individually in the estimation of esds in distances, angles and torsion angles; correlations between esds in cell parameters are only used when they are defined by crystal symmetry. An approximate (isotropic) treatment of cell esds is used for estimating esds involving l.s. planes.
Refinement. Refinement of F^2^ against ALL reflections. The weighted R-factor wR and goodness of fit S are based on F^2^, conventional R-factors R are based on F, with F set to zero for negative F^2^. The threshold expression of F^2^ > 2sigma(F^2^) is used only for calculating R-factors(gt) etc. and is not relevant to the choice of reflections for refinement. R-factors based on F^2^ are statistically about twice as large as those based on F, and R- factors based on ALL data will be even larger.

### Fractional atomic coordinates and isotropic or equivalent isotropic displacement parameters (Å^2^)

**Table:**

	*x*	*y*	*z*	*U*_iso_*/*U*_eq_	Occ. (<1)
P1A	0.11821 (2)	0.85263 (9)	0.07264 (5)	0.0284 (2)	0.5033 (10)
O1A	0.0996 (2)	0.6755 (7)	−0.0927 (3)	0.0445 (10)	0.5033 (10)
O2A	0.21408 (13)	0.8979 (6)	0.1073 (3)	0.0635 (12)	0.5033 (10)
O3A	0.05865 (9)	1.1130 (3)	0.1131 (2)	0.0508 (7)	0.5033 (10)
C1A	0.13426 (11)	0.6409 (4)	0.0532 (2)	0.0284 (7)	0.5033 (10)
C2A	0.12437 (10)	0.5761 (4)	−0.0329 (2)	0.0410 (6)	0.5033 (10)
C3A	0.13987 (15)	0.4212 (7)	−0.0534 (3)	0.0396 (16)	0.5033 (10)
H3A	0.1340	0.3806	−0.1117	0.048*	0.5033 (10)
C4A	0.16347 (14)	0.3302 (5)	0.0115 (3)	0.0595 (11)	0.5033 (10)
H4A	0.1740	0.2278	−0.0028	0.071*	0.5033 (10)
C5A	0.1721 (3)	0.3871 (7)	0.0983 (5)	0.0896 (14)	0.5033 (10)
H5A	0.1869	0.3213	0.1430	0.108*	0.5033 (10)
C6A	0.15860 (11)	0.5437 (4)	0.1188 (2)	0.0382 (7)	0.5033 (10)
H6A	0.1658	0.5843	0.1767	0.046*	0.5033 (10)
C7A	0.14947 (10)	0.8925 (3)	0.18335 (19)	0.0328 (6)	0.5033 (10)
C8A	0.19597 (13)	0.9198 (5)	0.1871 (3)	0.0487 (9)	0.5033 (10)
C9A	0.2214 (2)	0.9672 (7)	0.2657 (5)	0.0696 (17)	0.5033 (10)
H9A	0.2525	0.9845	0.2666	0.083*	0.5033 (10)
C10A	0.2007 (2)	0.9887 (5)	0.3428 (4)	0.0812 (17)	0.5033 (10)
H10A	0.2179	1.0208	0.3958	0.097*	0.5033 (10)
C11A	0.1542 (2)	0.9627 (5)	0.3423 (3)	0.0670 (14)	0.5033 (10)
H11A	0.1401	0.9774	0.3944	0.080*	0.5033 (10)
C12A	0.12902 (13)	0.9143 (4)	0.2622 (2)	0.0424 (7)	0.5033 (10)
H12A	0.0979	0.8962	0.2613	0.051*	0.5033 (10)
C13A	0.05989 (10)	0.8286 (4)	0.1004 (2)	0.0368 (7)	0.5033 (10)
C14A	0.03431 (14)	0.9685 (5)	0.1150 (3)	0.0478 (10)	0.5033 (10)
C15A	−0.01091 (16)	0.9581 (8)	0.1305 (5)	0.0642 (16)	0.5033 (10)
H15A	−0.0270	1.0515	0.1421	0.077*	0.5033 (10)
C16A	−0.03183 (13)	0.8085 (6)	0.1288 (4)	0.0763 (15)	0.5033 (10)
H16A	−0.0627	0.8052	0.1359	0.092*	0.5033 (10)
C17A	−0.00931 (19)	0.6586 (7)	0.1167 (6)	0.0678 (11)	0.5033 (10)
H17A	−0.0231	0.5574	0.1198	0.081*	0.5033 (10)
C18A	0.03710 (13)	0.6805 (5)	0.0990 (3)	0.0491 (10)	0.5033 (10)
H18A	0.0532	0.5881	0.0855	0.059*	0.5033 (10)
C19A	0.09955 (16)	0.6406 (6)	−0.1872 (3)	0.0579 (10)	0.5033 (10)
H19A	0.0864	0.7305	−0.2220	0.087*	0.5033 (10)
H19B	0.0820	0.5442	−0.2021	0.087*	0.5033 (10)
H19C	0.1302	0.6239	−0.2006	0.087*	0.5033 (10)
C20A	0.25882 (15)	0.9331 (7)	0.1125 (3)	0.0410 (6)	0.5033 (10)
H20A	0.2702	0.9051	0.0567	0.061*	0.5033 (10)
H20B	0.2749	0.8719	0.1607	0.061*	0.5033 (10)
H20C	0.2632	1.0473	0.1238	0.061*	0.5033 (10)
C21A	0.03439 (3)	1.25999 (10)	0.11792 (5)	0.0859 (15)	0.5033 (10)
H21A	0.0549	1.3502	0.1161	0.129*	0.5033 (10)
H21B	0.0206	1.2631	0.1731	0.129*	0.5033 (10)
H21C	0.0111	1.2667	0.0678	0.129*	0.5033 (10)
P1B	0.12322 (2)	0.74119 (9)	0.15377 (5)	0.0330 (2)	0.4967 (10)
O1B	0.16399 (2)	0.44061 (9)	0.10141 (7)	0.0576 (9)	0.4967 (10)
O2B	0.16390 (2)	0.92945 (9)	0.30307 (6)	0.0431 (6)	0.4967 (10)
O3B	0.02690 (3)	0.71548 (10)	0.13196 (9)	0.0623 (10)	0.4967 (10)
C1B	0.11971 (6)	0.65852 (15)	0.03978 (5)	0.0317 (9)	0.4967 (10)
C2B	0.14037 (6)	0.50871 (15)	0.02601 (5)	0.0404 (7)	0.4967 (10)
C3B	0.13712 (6)	0.43983 (16)	−0.05953 (5)	0.049 (2)	0.4967 (10)
H3B	0.1495	0.3373	−0.0669	0.059*	0.4967 (10)
C4B	0.11634 (11)	0.5190 (5)	−0.1325 (3)	0.0502 (9)	0.4967 (10)
H4B	0.1160	0.4736	−0.1896	0.060*	0.4967 (10)
C5B	0.0956 (3)	0.6672 (12)	−0.1218 (4)	0.0458 (14)	0.4967 (10)
H5B	0.0806	0.7207	−0.1712	0.055*	0.4967 (10)
C6B	0.09754 (11)	0.7356 (5)	−0.0366 (2)	0.0398 (7)	0.4967 (10)
H6B	0.0837	0.8357	−0.0300	0.048*	0.4967 (10)
C7B	0.17805 (10)	0.8463 (4)	0.15924 (19)	0.0283 (5)	0.4967 (10)
C8B	0.19367 (11)	0.9314 (4)	0.2379 (2)	0.0318 (6)	0.4967 (10)
C9B	0.23494 (15)	1.0121 (6)	0.2487 (3)	0.0418 (10)	0.4967 (10)
H9B	0.2442	1.0693	0.3012	0.050*	0.4967 (10)
C10B	0.26237 (11)	1.0052 (5)	0.1782 (3)	0.0456 (8)	0.4967 (10)
H10B	0.2907	1.0559	0.1854	0.055*	0.4967 (10)
C11B	0.2480 (2)	0.9209 (8)	0.0934 (3)	0.0656 (17)	0.4967 (10)
H11B	0.2652	0.9208	0.0450	0.079*	0.4967 (10)
C12B	0.20682 (16)	0.8418 (5)	0.0913 (3)	0.0316 (8)	0.4967 (10)
H12B	0.1974	0.7804	0.0404	0.038*	0.4967 (10)
C13B	0.08356 (10)	0.9123 (4)	0.1363 (2)	0.0348 (6)	0.4967 (10)
C14B	0.03738 (12)	0.8789 (6)	0.1299 (3)	0.0453 (9)	0.4967 (10)
C15B	0.00492 (17)	1.0010 (8)	0.1214 (5)	0.0577 (13)	0.4967 (10)
H15B	−0.0258	0.9751	0.1188	0.069*	0.4967 (10)
C16B	0.01844 (14)	1.1617 (6)	0.1170 (3)	0.0656 (12)	0.4967 (10)
H16B	−0.0032	1.2441	0.1098	0.079*	0.4967 (10)
C17B	0.06457 (16)	1.1998 (7)	0.1235 (4)	0.0678 (11)	0.4967 (10)
H17B	0.0741	1.3075	0.1219	0.081*	0.4967 (10)
C18B	0.09617 (11)	1.0742 (4)	0.1323 (3)	0.0440 (8)	0.4967 (10)
H18B	0.1269	1.0998	0.1356	0.053*	0.4967 (10)
C19B	0.1803 (2)	0.2818 (5)	0.0964 (4)	0.0896 (14)	0.4967 (10)
H19D	0.1903	0.2430	0.1560	0.134*	0.4967 (10)
H19E	0.2055	0.2806	0.0609	0.134*	0.4967 (10)
H19F	0.1565	0.2128	0.0690	0.134*	0.4967 (10)
C20B	0.17636 (17)	1.0245 (6)	0.3812 (3)	0.0588 (11)	0.4967 (10)
H20D	0.1524	1.0212	0.4195	0.088*	0.4967 (10)
H20E	0.1814	1.1350	0.3639	0.088*	0.4967 (10)
H20F	0.2038	0.9817	0.4130	0.088*	0.4967 (10)
C21B	−0.0196 (2)	0.6854 (9)	0.1278 (7)	0.0859 (15)	0.4967 (10)
H21D	−0.0245	0.5721	0.1389	0.129*	0.4967 (10)
H21E	−0.0340	0.7139	0.0692	0.129*	0.4967 (10)
H21F	−0.0323	0.7494	0.1726	0.129*	0.4967 (10)

### Atomic displacement parameters (Å^2^)

**Table:**

	*U*^11^	*U*^22^	*U*^33^	*U*^12^	*U*^13^	*U*^23^
P1A	0.0297 (4)	0.0262 (3)	0.0281 (4)	−0.0024 (3)	−0.0023 (3)	0.0026 (3)
O1A	0.0543 (19)	0.0487 (17)	0.027 (2)	0.0000 (13)	−0.0113 (18)	−0.0048 (19)
O2A	0.0242 (14)	0.087 (3)	0.078 (3)	−0.0219 (18)	0.0018 (14)	−0.006 (2)
O3A	0.0461 (14)	0.0345 (13)	0.0688 (18)	0.0072 (11)	−0.0076 (12)	−0.0080 (13)
C1A	0.0191 (17)	0.0299 (15)	0.0361 (15)	−0.0035 (12)	0.0023 (12)	−0.0003 (12)
C2A	0.0290 (11)	0.0477 (14)	0.0449 (15)	−0.0056 (10)	−0.0015 (10)	−0.0047 (12)
C3A	0.027 (3)	0.036 (2)	0.056 (3)	−0.003 (2)	0.005 (3)	−0.017 (2)
C4A	0.056 (2)	0.0375 (19)	0.082 (3)	0.0108 (16)	−0.007 (2)	−0.0184 (18)
C5A	0.116 (3)	0.0338 (17)	0.107 (3)	0.0211 (19)	−0.043 (2)	−0.0097 (19)
C6A	0.0366 (15)	0.0336 (16)	0.0415 (17)	−0.0010 (13)	−0.0092 (12)	−0.0011 (13)
C7A	0.0393 (15)	0.0248 (13)	0.0319 (14)	−0.0029 (11)	−0.0060 (11)	0.0028 (11)
C8A	0.046 (2)	0.042 (2)	0.053 (2)	−0.0104 (16)	−0.0172 (18)	−0.002 (2)
C9A	0.067 (4)	0.049 (3)	0.082 (4)	−0.015 (2)	−0.041 (3)	−0.004 (3)
C10A	0.130 (4)	0.042 (2)	0.058 (3)	−0.005 (3)	−0.053 (3)	−0.008 (2)
C11A	0.132 (4)	0.0335 (19)	0.031 (2)	0.011 (2)	−0.012 (2)	−0.0032 (16)
C12A	0.066 (2)	0.0300 (15)	0.0304 (15)	0.0059 (15)	0.0016 (14)	0.0025 (12)
C13A	0.0255 (13)	0.0363 (16)	0.0472 (17)	0.0038 (12)	−0.0016 (13)	−0.0010 (13)
C14A	0.036 (2)	0.041 (2)	0.063 (2)	0.0114 (17)	−0.0115 (16)	−0.0106 (19)
C15A	0.033 (3)	0.065 (3)	0.093 (4)	0.014 (2)	−0.001 (3)	−0.029 (3)
C16A	0.0260 (16)	0.079 (3)	0.125 (4)	0.0004 (18)	0.012 (2)	−0.023 (3)
C17A	0.0469 (18)	0.0447 (19)	0.112 (3)	0.0060 (14)	0.0080 (18)	−0.012 (2)
C18A	0.0349 (18)	0.042 (2)	0.072 (3)	−0.0057 (15)	0.0116 (17)	−0.0071 (19)
C19A	0.075 (3)	0.066 (3)	0.0310 (18)	−0.014 (2)	−0.0030 (17)	−0.0049 (17)
C20A	0.0290 (11)	0.0477 (14)	0.0449 (15)	−0.0056 (10)	−0.0015 (10)	−0.0047 (12)
C21A	0.054 (2)	0.050 (2)	0.152 (4)	0.0102 (16)	0.004 (2)	−0.025 (2)
P1B	0.0320 (4)	0.0339 (4)	0.0334 (4)	−0.0019 (3)	0.0054 (3)	0.0063 (3)
O1B	0.072 (2)	0.0230 (16)	0.0701 (19)	−0.0038 (14)	−0.0264 (16)	0.0008 (15)
O2B	0.0494 (14)	0.0543 (16)	0.0261 (12)	0.0026 (12)	0.0058 (11)	−0.0029 (11)
O3B	0.0374 (15)	0.0579 (19)	0.097 (3)	−0.0119 (13)	0.0295 (15)	−0.0223 (18)
C1B	0.0204 (19)	0.0321 (17)	0.0430 (19)	−0.0036 (13)	0.0049 (14)	−0.0003 (14)
C2B	0.0299 (14)	0.0284 (15)	0.061 (2)	−0.0059 (12)	−0.0030 (14)	−0.0048 (14)
C3B	0.031 (4)	0.044 (4)	0.070 (4)	−0.005 (3)	0.000 (3)	−0.019 (3)
C4B	0.0298 (15)	0.069 (2)	0.054 (2)	−0.0125 (15)	0.0124 (14)	−0.0253 (19)
C5B	0.037 (2)	0.065 (3)	0.033 (3)	0.003 (2)	−0.004 (2)	−0.008 (3)
C6B	0.0355 (15)	0.0464 (19)	0.0373 (17)	0.0073 (14)	0.0034 (13)	−0.0022 (15)
C7B	0.0272 (13)	0.0298 (14)	0.0267 (13)	0.0029 (12)	−0.0021 (11)	0.0033 (11)
C8B	0.0336 (16)	0.0348 (16)	0.0257 (14)	0.0069 (13)	−0.0026 (13)	0.0011 (13)
C9B	0.038 (2)	0.046 (3)	0.039 (2)	0.0000 (17)	−0.0068 (15)	−0.0147 (19)
C10B	0.0288 (14)	0.052 (2)	0.055 (2)	−0.0071 (14)	−0.0022 (13)	−0.0116 (17)
C11B	0.065 (3)	0.080 (3)	0.042 (2)	−0.003 (3)	−0.036 (2)	−0.022 (2)
C12B	0.032 (2)	0.0327 (19)	0.0288 (16)	0.0021 (15)	−0.0001 (13)	−0.0029 (13)
C13B	0.0293 (13)	0.0415 (16)	0.0347 (15)	0.0021 (12)	0.0076 (11)	0.0003 (13)
C14B	0.0299 (17)	0.053 (2)	0.055 (2)	0.0015 (17)	0.0109 (15)	−0.015 (2)
C15B	0.027 (2)	0.075 (3)	0.074 (3)	0.008 (2)	0.014 (2)	−0.014 (3)
C16B	0.045 (2)	0.075 (3)	0.076 (3)	0.025 (2)	0.006 (2)	−0.003 (2)
C17B	0.0469 (18)	0.0447 (19)	0.112 (3)	0.0060 (14)	0.0080 (18)	−0.012 (2)
C18B	0.0347 (15)	0.0408 (17)	0.056 (2)	0.0024 (13)	0.0034 (14)	0.0090 (15)
C19B	0.116 (3)	0.0338 (17)	0.107 (3)	0.0211 (19)	−0.043 (2)	−0.0097 (19)
C20B	0.073 (3)	0.067 (3)	0.038 (2)	0.004 (2)	0.0107 (19)	−0.013 (2)
C21B	0.054 (2)	0.050 (2)	0.152 (4)	0.0102 (16)	0.004 (2)	−0.025 (2)

### Geometric parameters (Å, °)

**Table:**

P1A—C1A	1.836 (4)	P1B—C1B	1.8258
P1A—C7A	1.833 (3)	P1B—C7B	1.831 (3)
P1A—C13A	1.829 (3)	P1B—C13B	1.831 (3)
O1A—C2A	1.362 (6)	O1B—C2B	1.3767
O1A—C19A	1.440 (4)	O1B—C19B	1.397 (4)
O2A—C20A	1.348 (5)	O2B—C8B	1.383 (3)
O2A—C8A	1.373 (6)	O2B—C20B	1.419 (4)
O3A—C14A	1.392 (5)	O3B—C14B	1.379 (5)
O3A—C21A	1.411 (3)	O3B—C21B	1.390 (6)
C1A—C2A	1.393 (5)	C1B—C2B	1.400
C1A—C6A	1.400 (5)	C1B—C6B	1.403 (3)
C2A—C3A	1.399 (6)	C2B—C3B	1.3914
C3A—C4A	1.356 (7)	C3B—C4B	1.357 (5)
C3A—H3A	0.93	C3B—H3B	0.93
C4A—C5A	1.375 (8)	C4B—C5B	1.381 (9)
C4A—H4A	0.93	C4B—H4B	0.93
C5A—C6A	1.392 (6)	C5B—C6B	1.386 (6)
C5A—H5A	0.93	C5B—H5B	0.93
C6A—H6A	0.93	C6B—H6B	0.93
C7A—C8A	1.388 (5)	C7B—C12B	1.395 (5)
C7A—C12A	1.394 (4)	C7B—C8B	1.402 (4)
C8A—C9A	1.379 (7)	C8B—C9B	1.383 (5)
C9A—C10A	1.375 (9)	C9B—C10B	1.400 (6)
C9A—H9A	0.93	C9B—H9B	0.93
C10A—C11A	1.390 (8)	C10B—C11B	1.465 (5)
C10A—H10A	0.93	C10B—H10B	0.93
C11A—C12A	1.396 (5)	C11B—C12B	1.377 (7)
C11A—H11A	0.93	C11B—H11B	0.93
C12A—H12A	0.93	C12B—H12B	0.93
C13A—C18A	1.391 (5)	C13B—C18B	1.386 (5)
C13A—C14A	1.406 (5)	C13B—C14B	1.386 (4)
C14A—C15A	1.385 (6)	C14B—C15B	1.385 (7)
C15A—C16A	1.375 (7)	C15B—C16B	1.385 (8)
C15A—H15A	0.93	C15B—H15B	0.93
C16A—C17A	1.421 (6)	C16B—C17B	1.392 (6)
C16A—H16A	0.93	C16B—H16B	0.93
C17A—C18A	1.437 (6)	C17B—C18B	1.389 (6)
C17A—H17A	0.93	C17B—H17B	0.93
C18A—H18A	0.93	C18B—H18B	0.93
C19A—H19A	0.96	C19B—H19D	0.96
C19A—H19B	0.96	C19B—H19E	0.96
C19A—H19C	0.96	C19B—H19F	0.96
C20A—H20A	0.96	C20B—H20D	0.96
C20A—H20B	0.96	C20B—H20E	0.96
C20A—H20C	0.96	C20B—H20F	0.96
C21A—H21A	0.96	C21B—H21D	0.96
C21A—H21B	0.96	C21B—H21E	0.96
C21A—H21C	0.96	C21B—H21F	0.96
			
C7A—P1A—C1A	101.47 (14)	O1B—C2B—C3B	124.3
C13A—P1A—C1A	101.57 (15)	O1B—C2B—C1B	115.3
C13A—P1A—C7A	102.02 (15)	C3B—C2B—C1B	120.4
C2A—O1A—C19A	117.8 (5)	C4B—C3B—C2B	121.44 (16)
C20A—O2A—C8A	113.4 (4)	C4B—C3B—H3B	119.3
C14A—O3A—C21A	117.6 (3)	C2B—C3B—H3B	119.3
C2A—C1A—C6A	117.8 (3)	C3B—C4B—C5B	119.8 (3)
C2A—C1A—P1A	118.5 (3)	C3B—C4B—H4B	120.1
C6A—C1A—P1A	123.5 (3)	C5B—C4B—H4B	120.1
O1A—C2A—C1A	114.8 (3)	C4B—C5B—C6B	119.4 (5)
O1A—C2A—C3A	124.5 (4)	C4B—C5B—H5B	120.3
C1A—C2A—C3A	120.7 (4)	C6B—C5B—H5B	120.3
C4A—C3A—C2A	120.0 (4)	C5B—C6B—C1B	122.1 (4)
C4A—C3A—H3A	120.0	C5B—C6B—H6B	118.9
C2A—C3A—H3A	120.0	C1B—C6B—H6B	118.9
C3A—C4A—C5A	120.9 (4)	C12B—C7B—C8B	116.9 (3)
C3A—C4A—H4A	119.5	C12B—C7B—P1B	124.3 (3)
C5A—C4A—H4A	119.5	C8B—C7B—P1B	118.7 (2)
C4A—C5A—C6A	119.6 (5)	C9B—C8B—O2B	123.1 (3)
C4A—C5A—H5A	120.2	C9B—C8B—C7B	122.8 (3)
C6A—C5A—H5A	120.2	O2B—C8B—C7B	114.1 (3)
C5A—C6A—C1A	120.8 (4)	C8B—C9B—C10B	118.1 (4)
C5A—C6A—H6A	119.6	C8B—C9B—H9B	121.0
C1A—C6A—H6A	119.6	C10B—C9B—H9B	121.0
C8A—C7A—C12A	117.8 (3)	C9B—C10B—C11B	122.2 (4)
C8A—C7A—P1A	117.6 (3)	C9B—C10B—H10B	118.9
C12A—C7A—P1A	124.2 (3)	C11B—C10B—H10B	118.9
O2A—C8A—C9A	123.3 (5)	C12B—C11B—C10B	114.4 (5)
O2A—C8A—C7A	115.1 (4)	C12B—C11B—H11B	122.8
C9A—C8A—C7A	121.6 (5)	C10B—C11B—H11B	122.8
C10A—C9A—C8A	119.9 (6)	C11B—C12B—C7B	125.5 (4)
C10A—C9A—H9A	120.1	C11B—C12B—H12B	117.3
C8A—C9A—H9A	120.1	C7B—C12B—H12B	117.3
C9A—C10A—C11A	120.5 (4)	C18B—C13B—C14B	117.1 (3)
C9A—C10A—H10A	119.7	C18B—C13B—P1B	124.9 (2)
C11A—C10A—H10A	119.7	C14B—C13B—P1B	117.9 (3)
C10A—C11A—C12A	118.8 (5)	O3B—C14B—C15B	123.5 (3)
C10A—C11A—H11A	120.6	O3B—C14B—C13B	114.4 (3)
C12A—C11A—H11A	120.6	C15B—C14B—C13B	122.0 (4)
C7A—C12A—C11A	121.4 (4)	C16B—C15B—C14B	119.7 (4)
C7A—C12A—H12A	119.3	C16B—C15B—H15B	120.2
C11A—C12A—H12A	119.3	C14B—C15B—H15B	120.2
C18A—C13A—C14A	116.7 (3)	C15B—C16B—C17B	119.8 (4)
C18A—C13A—P1A	124.0 (3)	C15B—C16B—H16B	120.1
C14A—C13A—P1A	119.0 (3)	C17B—C16B—H16B	120.1
C15A—C14A—O3A	124.6 (4)	C18B—C17B—C16B	118.9 (5)
C15A—C14A—C13A	121.4 (4)	C18B—C17B—H17B	120.5
O3A—C14A—C13A	114.0 (3)	C16B—C17B—H17B	120.5
C16A—C15A—C14A	119.5 (5)	C13B—C18B—C17B	122.5 (4)
C16A—C15A—H15A	120.3	C13B—C18B—H18B	118.8
C14A—C15A—H15A	120.3	C17B—C18B—H18B	118.8
C15A—C16A—C17A	124.1 (4)	O1B—C19B—H19D	109.5
C15A—C16A—H16A	117.9	O1B—C19B—H19E	109.5
C17A—C16A—H16A	117.9	H19D—C19B—H19E	109.5
C16A—C17A—C18A	112.7 (5)	O1B—C19B—H19F	109.5
C16A—C17A—H17A	123.7	H19D—C19B—H19F	109.5
C18A—C17A—H17A	123.7	H19E—C19B—H19F	109.5
C13A—C18A—C17A	125.3 (4)	O2B—C20B—H20D	109.5
C13A—C18A—H18A	117.4	O2B—C20B—H20E	109.5
C17A—C18A—H18A	117.4	H20D—C20B—H20E	109.5
C1B—P1B—C7B	100.11 (12)	O2B—C20B—H20F	109.5
C1B—P1B—C13B	100.60 (10)	H20D—C20B—H20F	109.5
C7B—P1B—C13B	101.19 (14)	H20E—C20B—H20F	109.5
C2B—O1B—C19B	118.9 (2)	O3B—C21B—H21D	109.5
C8B—O2B—C20B	116.4 (3)	O3B—C21B—H21E	109.5
C14B—O3B—C21B	113.3 (3)	H21D—C21B—H21E	109.5
C2B—C1B—C6B	116.71 (16)	O3B—C21B—H21F	109.5
C2B—C1B—P1B	118.9	H21D—C21B—H21F	109.5
C6B—C1B—P1B	124.34 (16)	H21E—C21B—H21F	109.5
			
C13A—P1A—C1A—C2A	87.8 (3)	C7B—P1B—C1B—C2B	85.86 (10)
C7A—P1A—C1A—C2A	−167.3 (3)	C13B—P1B—C1B—C2B	−170.61 (10)
C13A—P1A—C1A—C6A	−96.6 (3)	C7B—P1B—C1B—C6B	−93.9 (2)
C7A—P1A—C1A—C6A	8.4 (3)	C13B—P1B—C1B—C6B	9.6 (3)
C19A—O1A—C2A—C1A	161.8 (4)	C19B—O1B—C2B—C3B	−9.9 (3)
C19A—O1A—C2A—C3A	−17.4 (7)	C19B—O1B—C2B—C1B	171.4 (3)
C6A—C1A—C2A—O1A	178.4 (4)	C6B—C1B—C2B—O1B	176.5 (2)
P1A—C1A—C2A—O1A	−5.7 (5)	P1B—C1B—C2B—O1B	−3.3
C6A—C1A—C2A—C3A	−2.3 (5)	C6B—C1B—C2B—C3B	−2.20 (18)
P1A—C1A—C2A—C3A	173.6 (3)	P1B—C1B—C2B—C3B	177.99 (8)
O1A—C2A—C3A—C4A	−178.6 (5)	O1B—C2B—C3B—C4B	−174.7 (2)
C1A—C2A—C3A—C4A	2.2 (6)	C1B—C2B—C3B—C4B	3.85 (19)
C2A—C3A—C4A—C5A	0.7 (8)	C2B—C3B—C4B—C5B	−3.6 (6)
C3A—C4A—C5A—C6A	−3.5 (10)	C3B—C4B—C5B—C6B	1.9 (10)
C4A—C5A—C6A—C1A	3.3 (9)	C4B—C5B—C6B—C1B	−0.3 (10)
C2A—C1A—C6A—C5A	−0.4 (6)	C2B—C1B—C6B—C5B	0.5 (6)
P1A—C1A—C6A—C5A	−176.1 (5)	P1B—C1B—C6B—C5B	−179.7 (5)
C13A—P1A—C7A—C8A	−179.0 (3)	C1B—P1B—C7B—C12B	−4.2 (3)
C1A—P1A—C7A—C8A	76.4 (3)	C13B—P1B—C7B—C12B	−107.2 (3)
C13A—P1A—C7A—C12A	−6.2 (3)	C1B—P1B—C7B—C8B	178.8 (2)
C1A—P1A—C7A—C12A	−110.8 (3)	C13B—P1B—C7B—C8B	75.8 (3)
C20A—O2A—C8A—C9A	−2.4 (7)	C20B—O2B—C8B—C9B	4.0 (5)
C20A—O2A—C8A—C7A	177.0 (4)	C20B—O2B—C8B—C7B	−175.1 (3)
C12A—C7A—C8A—O2A	−179.4 (3)	C12B—C7B—C8B—C9B	1.7 (5)
P1A—C7A—C8A—O2A	−6.1 (5)	P1B—C7B—C8B—C9B	178.9 (3)
C12A—C7A—C8A—C9A	−0.1 (6)	C12B—C7B—C8B—O2B	−179.2 (3)
P1A—C7A—C8A—C9A	173.3 (4)	P1B—C7B—C8B—O2B	−2.0 (3)
O2A—C8A—C9A—C10A	179.2 (5)	O2B—C8B—C9B—C10B	179.8 (3)
C7A—C8A—C9A—C10A	−0.1 (8)	C7B—C8B—C9B—C10B	−1.2 (6)
C8A—C9A—C10A—C11A	0.1 (8)	C8B—C9B—C10B—C11B	2.2 (7)
C9A—C10A—C11A—C12A	0.1 (7)	C9B—C10B—C11B—C12B	−3.7 (8)
C8A—C7A—C12A—C11A	0.3 (5)	C10B—C11B—C12B—C7B	4.4 (8)
P1A—C7A—C12A—C11A	−172.5 (3)	C8B—C7B—C12B—C11B	−3.6 (6)
C10A—C11A—C12A—C7A	−0.3 (6)	P1B—C7B—C12B—C11B	179.4 (4)
C7A—P1A—C13A—C18A	−107.9 (3)	C1B—P1B—C13B—C18B	−104.3 (3)
C1A—P1A—C13A—C18A	−3.4 (4)	C7B—P1B—C13B—C18B	−1.7 (3)
C7A—P1A—C13A—C14A	79.2 (3)	C1B—P1B—C13B—C14B	78.1 (3)
C1A—P1A—C13A—C14A	−176.3 (3)	C7B—P1B—C13B—C14B	−179.2 (3)
C21A—O3A—C14A—C15A	−6.9 (7)	C21B—O3B—C14B—C15B	−2.0 (7)
C21A—O3A—C14A—C13A	173.7 (3)	C21B—O3B—C14B—C13B	178.4 (5)
C18A—C13A—C14A—C15A	2.3 (7)	C18B—C13B—C14B—O3B	178.4 (3)
P1A—C13A—C14A—C15A	175.8 (5)	P1B—C13B—C14B—O3B	−3.9 (4)
C18A—C13A—C14A—O3A	−178.2 (4)	C18B—C13B—C14B—C15B	−1.2 (6)
P1A—C13A—C14A—O3A	−4.8 (5)	P1B—C13B—C14B—C15B	176.5 (4)
O3A—C14A—C15A—C16A	178.4 (5)	O3B—C14B—C15B—C16B	−177.9 (5)
C13A—C14A—C15A—C16A	−2.2 (9)	C13B—C14B—C15B—C16B	1.6 (8)
C14A—C15A—C16A—C17A	3.6 (11)	C14B—C15B—C16B—C17B	−1.7 (9)
C15A—C16A—C17A—C18A	−4.8 (10)	C15B—C16B—C17B—C18B	1.4 (8)
C14A—C13A—C18A—C17A	−4.1 (7)	C14B—C13B—C18B—C17B	0.9 (6)
P1A—C13A—C18A—C17A	−177.1 (5)	P1B—C13B—C18B—C17B	−176.7 (4)
C16A—C17A—C18A—C13A	5.1 (9)	C16B—C17B—C18B—C13B	−1.0 (7)

### Hydrogen-bond geometry (Å, °)

**Table:**

*D*—H···*A*	*D*—H	H···*A*	*D*···*A*	*D*—H···*A*
C21A—H21C···Cg1^i^	0.96	2.83	3.662 (3)	145

Symmetry codes: (i) *x*+1/2, *y*+5/2, *z*.
